# Atelectasis, Shunt, and Worsening Oxygenation Following Reduction of Respiratory Rate in Healthy Pigs Undergoing ECMO: An Experimental Lung Imaging Study

**DOI:** 10.3389/fphys.2021.663313

**Published:** 2021-04-09

**Authors:** Elena Spinelli, Giulia Colussi, Gaia Dal Santo, Eleonora Scotti, Ines Marongiu, Erica Garbelli, Alessandra Mazzucco, Daniele Dondossola, Raquel Maia, Michele Battistin, Osvaldo Biancolilli, Lorenzo Rosso, Stefano Gatti, Tommaso Mauri

**Affiliations:** ^1^Department of Anesthesia, Critical Care and Emergency, Fondazione Istituto di Ricovero e Cura a Carattere Scientifico (IRCCS) Ca' Granda Ospedale Maggiore Policlinico, Milan, Italy; ^2^Department of Pathophysiology and Transplantation, University of Milan, Milan, Italy; ^3^Thoracic Surgery and Lung Transplantation Unit, Fondazione Istituto di Ricovero e Cura a Carattere Scientifico (IRCCS) Ca' Granda Ospedale Maggiore Policlinico, Milan, Italy; ^4^General and Liver Transplant Surgery Unit, Fondazione Istituto di Ricovero e Cura a Carattere Scientifico (IRCCS) Ca' Granda Ospedale Maggiore Policlinico, Milan, Italy; ^5^Department of Intensive Care Medicine, Hospital Professor Doutor Fernando Fonseca, Amadora, Portugal; ^6^Center for Preclinical Research, Fondazione Istituto di Ricovero e Cura a Carattere Scientifico (IRCCS) Ca' Granda Ospedale Maggiore Policlinico, Milan, Italy

**Keywords:** extracorporeal membrane oxygenation, respiratory rate, atelectasis, shunt, expiratory time

## Abstract

**Rationale:** Reducing the respiratory rate during extracorporeal membrane oxygenation (ECMO) decreases the mechanical power, but it might induce alveolar de-recruitment. Dissecting de-recruitment due to lung edema vs. the fraction due to hypoventilation may be challenging in injured lungs.

**Objectives:** We characterized changes in lung physiology (primary endpoint: development of atelectasis) associated with progressive reduction of the respiratory rate in healthy animals on ECMO.

**Methods:** Six female pigs underwent general anesthesia and volume control ventilation (Baseline: PEEP 5 cmH_2_O, Vt 10 ml/kg, I:E = 1:2, FiO_2_ 0.5, rate 24 bpm). Veno-venous ECMO was started and respiratory rate was progressively reduced to 18, 12, and 6 breaths per minute (6-h steps), while all other settings remained unchanged. ECMO blood flow was kept constant while gas flow was increased to maintain stable PaCO_2_.

**Measurements and Main Results:** At Baseline (without ECMO) and toward the end of each step, data from quantitative CT scan, electrical impedance tomography, and gas exchange were collected. Increasing ECMO gas flow while lowering the respiratory rate was associated with an increase in the fraction of non-aerated tissue (i.e., atelectasis) and with a decrease of tidal ventilation reaching the gravitationally dependent lung regions (*p* = 0.009 and *p* = 0.018). Intrapulmonary shunt increased (*p* < 0.001) and arterial PaO_2_ decreased (*p* < 0.001) at lower rates. The fraction of non-aerated lung was correlated with longer expiratory time spent at zero flow (*r* = 0.555, *p* = 0.011).

**Conclusions:** Progressive decrease of respiratory rate coupled with increasing CO_2_ removal in mechanically ventilated healthy pigs is associated with development of lung atelectasis, higher shunt, and poorer oxygenation.

## Introduction

Veno-venous extracorporeal membrane oxygenation (ECMO) is a rescue strategy for patients with severe acute respiratory distress syndrome (ARDS) not responsive to conventional positive pressure ventilation (Combes et al., 2020). In the last few years, the use of ECMO increased worldwide since the H1N1 epidemic in 2009 and following publication of large clinical trials and observational analyses showing positive impact on mortality (Peek et al., 2009; Combes et al., 2018). ECMO support allows a reduction in mechanical ventilation load. Indeed, during ECMO, gas exchange becomes almost independent from the applied ventilation, and the latter can be reduced drastically, enhancing lung rest (Pesenti et al., 1982). Even though decreased ventilation is key to ECMO success and lung protection, physiological data supporting specific strategies are scant and published clinical trials implemented “ultra-protective ventilation” with very different settings (Gattinoni et al., 1986; Terragni et al., 2009; Bein et al., 2013; Combes et al., 2019).

In the present study, we focused on the physiological effects of decreasing respiratory rate. In usual clinical management of severe ARDS patients, respiratory rate is gradually reduced after start of ECMO but with highly variable targets (Spinelli et al., 2020); large clinical trials used rates ranging between 24 and 5 breaths per minute (Pesenti et al., 1982; Gattinoni et al., 1986; Peek et al., 2009; Terragni et al., 2009; Bein et al., 2013; Combes et al., 2018, 2019), and only two animal studies reported some physiological benefits of reduced respiratory rate during extracorporeal support, but rates differed widely, averaging 14 vs. 5 breaths per minute (Grasso et al., 2014; Araos et al., 2019). Thus, the physiological targets to guide the reduction of respiratory rate during ECMO are still unclear.

Lowering the respiratory rate leads to a reduction in the mechanical power applied to the lungs, and any decrease should be associated with improved lung protection (Marini et al., 2020). This is true only if all other determinants of ventilator-induced lung injury (VILI) remain stable (Marini et al., 2020). By contrast, previous experimental study described development of atelectasis when expiration becomes longer than 4 s (Neumann et al., 1998), and this might increase ventilation heterogeneity (Mauri et al., 2013), lung strain (Bellani et al., 2011), and atelectrauma (Caironi et al., 2010). Moreover, to maintain stable gas exchange, lower respiratory support requires higher extracorporeal oxygenation and CO_2_ removal, which might be associated with altered physiology (e.g., increased intrapulmonary shunt; Fanelli et al., 2016; Spinelli et al., 2020).

The aim of this study was to describe the physiological effects of the progressive reduction of the respiratory rate and concomitant increase of extracorporeal CO_2_ removal during ECMO in terms of non-aerated lung fraction measured by CT scan (primary endpoint), ventilation maldistribution, increased shunt, and hypoxemia due to low values of the respiratory exchange ratio (RER) of the natural lung. Since dissecting atelectasis caused by the compressive forces of lung edema vs. those due to hypoventilation may be challenging in injured lungs, we took a step backward and studied progressive reduction of respiratory rate and increased CO_2_ removal in a large animal ECMO model with healthy lungs.

## Materials and Methods

The study was approved by the Italian Ministry of Health (protocol n. 749/2019) and conducted according to the European Directive 2010/63/EU on the protection of animals used for scientific purposes and Italian legislative decree 26/2014. The research protocol was approved by the Institutional Animal Care Committee.

### Anesthesia, Animal Preparation, and Instrumentation

In compliance with local recommendations, pigs arrived at the experimental facility the day before the start of the study and fasted for 24 h with free access to water. Six healthy female pigs (40 ± 4 kg) were sedated by intramuscular injection of medetomidine 0.025 mg/kg and tiletamine/zolazepam 5 mg/kg. Then, an auricular vein was cannulated and, after administration of ceftriaxone 1 g and tramadol 50 mg, continuous intravenous (IV) infusion of Propofol was titrated to maintain the animal on spontaneous breathing and SpO_2_ 100% while oxygen was provided via face mask. Surgical tracheostomy was performed in the supine position under additional local anesthesia (Lidocaine 2%). After endotracheal tube was inserted through the tracheostomy and fixed, mechanical ventilation was started (see Baseline ventilation settings below) and general anesthesia was maintained by IV Propofol 5–10 mg/kg/h, Medetomidine 2.5–10.0 μg/kg/h, and Pancuronium bromide 0.3–0.5 mg/kg/h. Depth of anesthesia was adjusted to ensure no sign of distress, such as unexplained tachycardia, arterial hypertension, and horripilation. Ringer lactate was administered at 100 ml/h during surgery and along the whole study, unless otherwise indicated by hemodynamic requirements (see the hemodynamic protocol below). Ceftriaxone 1 g IV and tramadol 50 mg IV were repeated after 12 h.

Vascular accesses were obtained by surgical exposure. An arterial catheter (Seldicath, 5 Fr and 8 cm, Prodimed, France) was inserted in the left common carotid artery. A three-lumen central venous catheter (Arrow, 7 Fr, Teleflex, Ireland) and a pulmonary artery catheter (Swan Ganz, 5 Fr, Edwards, USA) were inserted and advanced in the left external jugular vein. Positioning of pulmonary artery catheter was achieved by direct visualization of pulmonary artery and wedge pressures.

An esophageal balloon catheter (5 Fr, Cooper Surgical, CT, USA) was inserted and inflated with the recommended volume of air. Correct positioning and calibration were confirmed by the standard occlusion test with external compressions.

### Baseline Ventilation Settings and Data Collection

During the whole experiment, apart from surgical procedures, animals were kept prone.

From tracheostomy to the end of Baseline data collection, volume-controlled mechanical ventilation (Evita XL, Drager, Germany) was set as follows:

- Fraction of inspired oxygen (FiO_2_) = 0.5
- Tidal volume (Vt) = 10 ml/kg body weight
- Respiratory rate (RR) = 24 breaths per minute
- Positive end-expiratory pressure (PEEP) = 5 cmH_2_O
- Inspiratory to expiratory time ratio (I:E) = 1:2

A heat and moisture exchange filter was part of the ventilator circuit.

Data from respiratory mechanics, hemodynamics, arterial, and mixed venous blood gas analysis, volumetric capnography (Respironics NM3 monitor, Philips, The Netherlands), and quantitative CT scan (Lightspeed, General Electric, USA) were collected at the end of the instrumentation phase (Baseline).

The variables collected were as follows:

- Respiratory mechanics: mean airway pressure (mPaw), tidal volume (Vt), plateau airway pressure (Pplat), total PEEP (PEEPtot), and change between inspiratory and expiratory esophageal pressure (ΔPes) by 3-s inspiratory and expiratory holds. From these, static respiratory system compliance (Crs) was calculated as Vt/(Pplat – PEEPtot), lung compliance (Clung) as Vt/((Pplat – PEEPtot) – ΔPes), and chest wall compliance (Ccw) as Vt/ΔPes (Mauri et al., 2016b). We also calculated mechanical power per minute by standard formula (Marini et al., 2020). Finally, we recorded tracings of airway pressure and flow and we measured the time spent by the respiratory system at zero flow during expiration (T_EXP_ at zero flow: the time between the zero expiratory flow and the start of inspiratory flow for the next breath), as the average of 5 breaths.
- Hemodynamics: mean arterial pressures (MAP), mean pulmonary artery pressures (PAPm), pulmonary capillary wedge pressure (WP) at end expiration, cardiac output (CO) via thermodilution technique (Vigilance, Baxter Edwards Critical Care, Edwards E6 Lifesciences, USA), heart rate (HR), central venous pressure (CVP) at end expiration, and mixed venous oxygen saturation (SvO_2_).
- Blood gas values: arterial pH, PaCO_2_, PaO_2_, Base Excess (BE), and lactates. Oxygen consumption through the natural lung (VO_2NL_) and intrapulmonary shunt calculated with Riley's method were also calculated from mixed venous and arterial blood gases by standard formulas (Zanella et al., 2016; Radermacher et al., 2017).
- Volumetric capnography: CO_2_ elimination by the natural lung (VCO_2NL_); alveolar partial pressure of oxygen (PAO_2_) calculated as:

$$\begin{array}{l} \text{PA}{\text{O}}_{2}=\text{Fi}{\text{O}}_{2}*\left({\text{P}}_{\text{atm}}-\text{PH}2\text{O}\right)-\text{PaC}{\text{O}}_{2}/\text{RE}{\text{R}}_{\text{NL}}\\ +\text{Fi}{\text{O}}_{2}*\text{PaC}{\text{O}}_{2}*\left(1-\text{E}{\text{R}}_{\text{NL}}\right)/\text{RE}{\text{R}}_{\text{NL}} \end{array}$$

Where RER _NL_ is the respiratory exchange ratio of the natural lung and was calculated as (Dickstein, 2020):

$$\begin{array}{l} \text{RE}{\text{R}}_{\text{NL}}=\text{VC}{\text{O}}_{2\text{NL}}/\text{V}{\text{O}}_{2\text{NL}} \end{array}$$

- CT scan: chest CT scans (Lightspeed®, General Electric, USA) were acquired during a respiratory hold performed at end expiration. Acquired images were processed offline for quantitative analysis, as previously described (Gattinoni et al., 2006). Briefly, lung boundaries were manually drawn on each slice and analyzed using a dedicated software program (Maluna 3.17, Göttingen, Germany). After processing each slice of a series, total lung weight expressed in grams of tissue was calculated by standard formulas and frequency distribution of lung CT numbers expressed in Hounsfield units (HUs) was computed. From this, lung units were classified as non-aerated (density > −100 HU), poorly aerated (−100 to −500 HU), and normally aerated (−500 to −900 HU). The percentages of non-aerated, poorly aerated, and normally aerated tissue were measured both for the whole lungs and for non-dependent (from halfway of the lungs up) and dependent (from halfway down) regions.
- Electrical impedance tomography (EIT): EIT data (Pulmovista, Drager, Lubeck, Germany) were continuously recorded for 2–3 min, during which end-expiratory and end-inspiratory holds were performed. From offline analysis, we measured the tidal ventilation distribution and the regional respiratory system compliances in two equal-size regions (non-dependent from halfway up and dependent from halfway down), as previously described (Mauri et al., 2016a; Scaramuzzo et al., 2020a).

### Extracorporeal Membrane Oxygenation

After Baseline data collection, animals were turned supine and veno-venous ECMO was started. ECMO circuit consisted of draining line, pump, return line (heparin-coated 3/8 polyvinylchloride circuit and Bio-pump BPX-80, Medtronic Italia SpA, Milan, Italy), membrane lung (EOS ECMO PMP oxygenator, Livanova, London, UK), return line heater, oxygen supply, and pressure transducer (positioned on the draining line) and was primed with balanced solution at a controlled temperature of 38°C. The left iliac and right external jugular veins were surgically cannulated (wire-reinforced venous cardiopulmonary bypass cannula, 18 Fr 36 cm, Sorin Group Italia srl, Mirandola, Italy) after an IV heparin bolus of 80 UI/kg, and ECMO was started by a gradual increase of blood flow (BF) up to 1.5 L/min. Then, heparin infusion was started at 40 UI/kg/h and titrated to obtain an Activated Coagulation Time of 180–210 s, measured every 1–2 h.

Once stable, animals were turned prone again.

### Study Protocol

After start of ECMO and return to the prone position, ventilation continued with the abovementioned settings (FiO_2_ = 0.5, Vt = 10 ml/kg, PEEP = 5 cmH_2_O, I:E = 1:2) and RR was promptly decreased to 18 bpm for 6 h. BF was maintained at 1.5 L/min for the whole study and ECMO sweep 100% oxygen gas flow (GF) was increased to maintain stable PaCO_2_ at values equal to Baseline ± 5 mmHg (arterial blood gases were performed every 30 min and GF adjusted by 0.5 L/min steps until stability for two subsequent measures). Toward the end of the RR 18 time period, data collection was performed again (see ECMO data collection below). Then, respiratory rate was decreased to 12 bpm for 6 h, leaving all other ventilation settings unchanged and adjusting ECMO GF as described above. Toward the end of the 6-h RR 12 period, ECMO data collection was performed again (see below). Finally, respiratory rate was reduced to 6 for the last 6 h, ECMO GF was adjusted to obtain stable PaCO_2_, and ECMO data collection was repeated toward the end. All animals completed the protocol.

Toward the end of each 6-h study phase, all the abovementioned Baseline data collection was repeated. The following relevant data on ECMO support were collected, too:

- Extracorporeal CO_2_ elimination (VCO_2__ML_) was measured by measuring the fraction of CO_2_ within the gas exiting the ECMO membrane lung and multiplying this by the actual ECMO GF.
- Oxygen consumption through the ECMO membrane lung (VO_2ML_) was calculated from pre- and post-lung blood gas analyses by standard formulas (Zanella et al., 2016).
- The RER of the membrane lung was calculated as:

(1) $$\begin{array}{r} {\text{RER}}_{\text{ML}}={\text{VCO}}_{2\text{ML}}/{\text{VO}}_{2\text{ML}} \end{array}$$

### Hemodynamic Protocol

Balanced electrolytes solution and norepinephrine were infused according to a standardized protocol, with a target of MAP above 60 mmHg. Every step of the protocol was applied only if the preceding one failed. If MAP was <60 mmHg:

- Balanced solution (Ringer) 250 ml bolus was administered, and infusion restarted at 150 ml/h;
- If MAP remained <60 mmHg, solution bolus was repeated and infusion was continued at 150 ml/h;
- If not responding to fluids, norepinephrine was started and titrated to obtain MAP >60 mmHg, with infusion at 150 ml/h.

When MAP rose above 70 mmHg, hemodynamic support was de-escalated according to the same protocol.

### Statistical Analysis

Study sample size was similar to previous similar studies (Pesenti et al., 1982; Neumann et al., 1998; Grasso et al., 2014). We calculated that six animals would have allowed us to detect an increase in the fraction of non-aerated tissue measured by CT scan with power of 0.8, alpha 0.05, and very large effect size (1.5). Data are shown as mean ± standard deviation. Comparisons between variables at each study time point were performed by one-way repeated measures ANOVA. Comparisons of non-aerated lung fractions at each time point in the non-dependent and dependent lung regions were performed by two-way repeated measures ANOVA. Holm–Sidak test was applied for *post-hoc* analyses. Correlations between variables were tested by Pearson's coefficient. Statistical significance was defined by *p* < 0.05 (two-tailed). Statistical analysis was performed using Sigma Plot 11.0 (Systat Software Inc., CA, USA).

## Results

### Progressive Reduction of Respiratory Rate and ECMO Support

Respiratory rate decreased from 24 to 6 bpm while CO_2_ removal (*p* < 0.001) gradually increased. Extracorporeal oxygenation (*p* < 0.001) increased after ECMO start and then remained stable (Table 1). ECMO provided around 40% of total VO_2_, with minimal differences between study steps, while the ECMO VCO_2_ increased progressively with the reduction of the respiratory rate, up to around 60% of total VCO_2_ at 6 breaths per minute (Table 1). Arterial partial pressure of CO_2_ remained stable throughout all study phases (*p* = 0.146; Table 1).

**Table 1:** Physiological effects of progressive reduction of respiratory rate and increase of extracorporeal CO_2_ removal during ECMO support—Part 1.

**Variables^°^**	**RR 24 Baseline**	**RR 18 + ECMO**	**RR 12 + ECMO**	**RR 6 + ECMO**	***P*-value^#^**
**ECMO**					
VCO_2__NL_, % total	100 ± 0	82 ± 7*	68 ± 13*	39 ± 7*	**<0.001**
VCO_2__ML_, % total	0 ± 0	18 ± 7*	32 ± 13*	61 ± 7*	**<0.001**
VO_2__NL_, % total	100 ± 0	60 ± 11*	59 ± 9*	62 ± 7*	**<0.001**
VO_2__ML_, % total	0 ± 0	40 ± 11*	41 ± 9*	38 ± 7*	**<0.001**
**Gas exchange**					
PaCO_2_, mmHg	34.6 ± 3.8	30.7 ± 2.7	33.1 ± 4.0	32.4 ± 2.3	0.146
PaO_2_/FiO_2_	526 ± 46	501 ± 49	509 ± 35	436 ± 46*	**<0.001**
**Computed tomography scan**					
Non-aerated lung (atelectasis), %	3 ± 2	3 ± 2	6 ± 4	12 ± 9*	**0.009**
Poorly-aerated lung, %	39 ± 7	39 ± 11	44 ± 11	46 ± 12	0.270
Normally-aerated lung, %	58 ± 8	58 ± 12	50 ± 14	42 ± 11*	**0.011**
Total lung weight, g	661 ± 119	647 ± 125	632 ± 95	695 ± 106	0.260
**Electrical impedance tomography**					
Vt _NDep_, %	29 ± 5	29 ± 6	34 ± 10	38 ± 11*	**0.018**
Vt _Dep_, %	71 ± 5	71 ± 6	66 ± 10	62 ± 11*	**0.018**
Crs _NDep_, ml/cmH_2_O	12 ± 2	9 ± 2	11 ± 3	12 ± 3	0.127
Crs _Dep_, ml/cmH_2_O	30 ± 8	24 ± 3*	23 ± 5*	20 ± 6*	**0.012**

# *One-way RM ANOVA*.

* *p < 0.05 Post-Hoc Holm–Sidak method vs. RR 24 Baseline value*.

° *ECMO, extracorporeal membrane oxygenation; VCO_2__NL_, VCO_2_ natural lung; VCO_2__ML_, VCO_2_ membrane lung; VO_2__NL_, VO_2_ natural lung; VO_2__ML_, VO_2_ membrane lung; PaCO_2_, arterial partial pressure of carbon dioxide; PaO_2_, arterial partial pressure of oxygen; FiO_2_, inspiratory fraction of oxygen; Vt _NDep_, tidal volume, non-dependent lung region; Vt _Dep_, tidal volume, dependent lung region; Crs _NDep_, respiratory system compliance, non-dependent region; Crs _Dep_, respiratory system compliance, dependent region. Significant p-values are presented in bold characters*.

### Effects of Lower Respiratory Rate and Higher Extracorporeal CO_2_ Removal by ECMO

**Figure 1:**
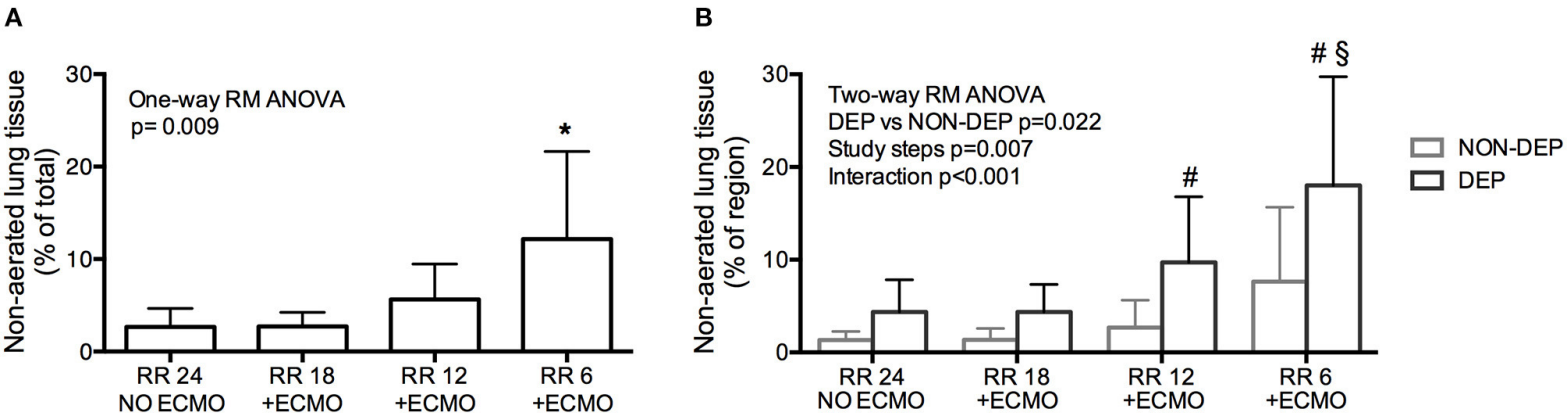
Development of atelectasis (non-aerated lung tissue) in healthy pigs undergoing Extracorporeal Membrane Oxygenation (ECMO) with progressive decrease of respiratory rate. **(A)** Development of atelectasis as increase of the % of Non-aerated lung tissue. **(B)** Regional atelectasis as increase of the % of non-aerated lung tissue in gravitationally dependent and non-dependent regions. *p < 0.05 *Post-Hoc* Holm–Sidak method vs. Baseline. ^#^*p* < 0.05 *Post-Hoc* Holm–Sidak method DEP vs. NON-DEP within study steps. ^§^*p* < 0.05 *Post-Hoc* Holm–Sidak method vs. Baseline within region (DEP).

The percentage of collapsed non-aerated lung tissue in the whole lungs measured by CT scan (i.e., lung atelectasis) significantly increased at lower respiratory rate and higher ECMO CO_2_ extraction (Figure 1A and Table 1). Development of atelectasis was mirrored by a reduction of the normally aerated lung tissue (*p* = 0.011), without change in total lung weight (i.e., no lung edema) measured by CT scan (*p* = 0.260; Table 1).

**Figure 2:**
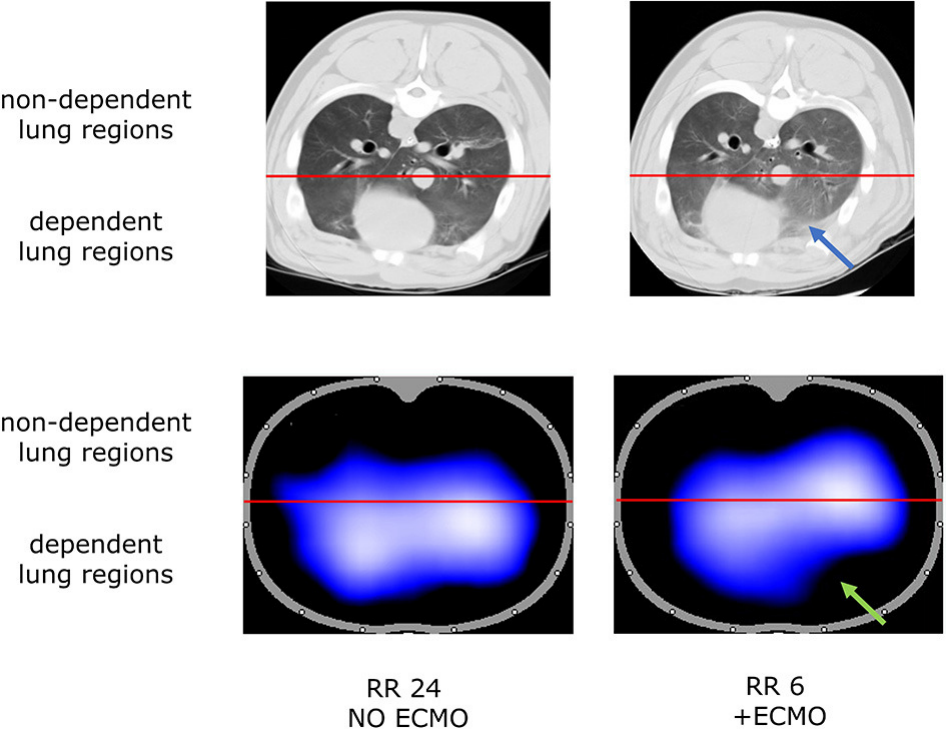
Representative CT scan (aeration at end expiration, **Top**) and Electrical Impedance Tomography (ventilation at end-inspiration, **Bottom**) images during RR 24 and RR 6 step from one representative animal. Blue arrow indicates lung collapse at CT scan; green arrow indicates the correspondent loss of aeration detected by EIT analysis.

The increase in non-aerated compartment measured by CT scan was more pronounced in the gravitationally dependent lung regions (Dep vs. NDep *p* = 0.022, study steps *p* = 0.007, interaction *p* < 0.001; Figure 1B). EIT data showed that collapse of the dependent lung caused a reduction of regional tidal volume at lower respiratory rate (*p* = 0.018), likely caused by decreased local respiratory system compliance (*p* = 0.012; Table 1). Figure 2 shows CT and EIT images for RR 24 and RR 6 study phase from a representative animal.

**Figure 3:**
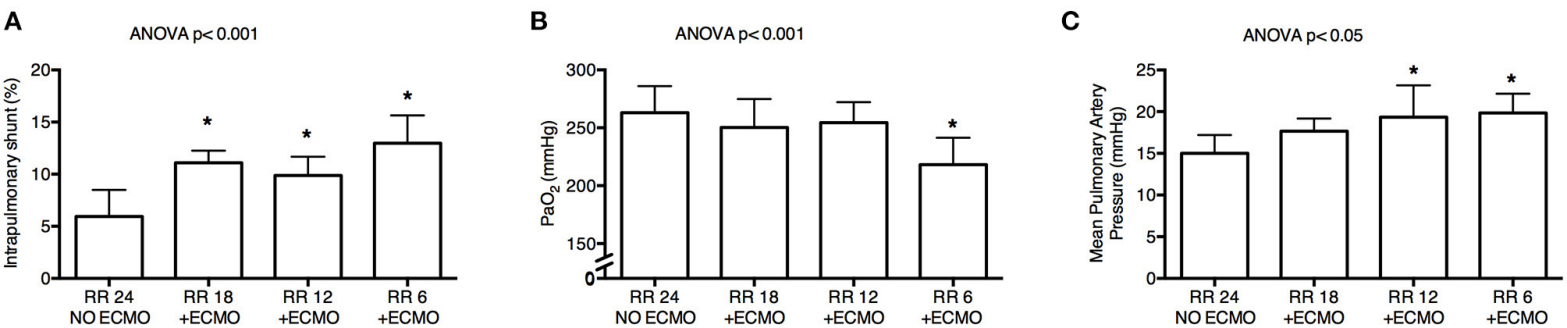
Physiological effects of lower respiratory rate and higher extracorporeal CO_2_ removal by ECMO. Progressive decrease of respiratory rate induced significant increase of intrapulmonary shunt calculated with Riley's method **(A)**, poorer systemic oxygenation **(B)**, and higher mean pulmonary artery pressure **(C)**. RR, respiratory rate; ECMO, extracorporeal membrane oxygenation; PaO_2_, arterial partial pressure of oxygen. *p < 0.05 *Post-Hoc* Holm–Sidak method vs. Baseline.

Progressive decrease of respiratory rate and increase of CO_2_ removal by ECMO were associated with increased intrapulmonary shunt (*p* < 0.001; Figure 3A) and with decreased PaO_2_ (*p* < 0.001; Figure 3B). FiO_2_ remained unchanged as per study protocol. Moreover, there was a reduction in arterial blood pH (*p* = 0.004) with decrease in base excess (*p* < 0.001) and bicarbonates (*p* < 0.001), possibly due to metabolic compensation for some respiratory alkalosis at Baseline (Table 2).

**Table 2:** Physiological effects of progressive reduction of respiratory rate and increase of extracorporeal CO_2_ removal during ECMO support—Part 2.

**Variables^°^**	**RR24 Baseline**	**RR18 + ECMO**	**RR12 + ECMO**	**RR6 + ECMO**	***P*-value^#^**
**Respiratory mechanics**					
Pplat, cmH_2_O	14 ± 1	17 ± 2*	17 ± 1*	17 ± 1*	**<0.001**
mPaw, cmH_2_O	9 ± 1	10 ± 1	9 ± 2	8 ± 1	0.183
Mechanical power, J/min	9.7 ± 1.0	8.8 ± 1.7	5.4 ± 0.8*	3.4 ± 0.7*	**<0.001**
T_EXP_ at zero flow, s	0.1 ± 0.2	0.4 ± 0.3	1.2 ± 0.5*	4.3 ± 0.8*	**<0.001**
Crs, ml/cmH_2_O	44 ± 6	34 ± 3*	34 ± 4*	34 ± 3*	**<0.001**
Ccw, ml/cmH_2_O	90 ± 14	74 ± 14	67 ± 18*	62 ± 9*	**0.014**
Clung, ml/cmH_2_O	88 ± 26	68 ± 24	77 ± 27	81 ± 26	0.475
Vt/EELV ratio	0.54 ± 0.06	0.52 ± 0.08	0.63 ± 0.16	0.62 ± 0.15	0.063
**Hemodynamics**					
MAP, mmHg	106 ± 8	116 ± 15	109 ± 9	101 ± 24	0.450
HR, bpm	93 ± 7	104 ± 14	98 ± 21	96 ± 21	0.681
PAPm, mmHg	15 ± 2	18 ± 2	19 ± 3*	20 ± 2*	**0.036**
PCWP, mmHg	6 ± 2	6 ± 1	6 ± 2	8 ± 2	0.265
CO, L/min	5.2 ± 0.5	4.7 ± 0.4	4.4 ± 0.8	4.6 ± 0.6	0.228
CVP, mmHg	2 ± 2	3 ± 1	3 ± 3	5 ± 2	0.119
SvO_2_, %	69 ± 4	80 ± 6	77 ± 5	76 ± 7	**0.008**
**Arterial acid–base balance**					
pH	7.54 ± 0.03	7.55 ± 0.04	7.50 ± 0.04*	7.49 ± 0.02*	**0.004**
HCO3^−^, mmol/L	29.6 ± 1.7	26.8 ± 2.1*	26.5 ± 1.4*	24.7 ± 1.8*	**<0.001**
BE, mmol/L	10.5 ± 10.3	8.2 ± 10.9*	7.4 ± 11.1*	5.7 ± 10.2*	**<0.001**
Lac, mmol/L	1.0 ± 0.3	0.7 ± 0.2	0.7 ± 0.4	0.6 ± 0.3	0.066

# *One-way RM ANOVA*.

* *p < 0.05 Post-Hoc Holm–Sidak method vs. RR 24 Baseline value*.

° *Pplat, plateau pressure; mPaw, mean airway pressure; T_EXP_ at zero flow, expiratory time at zero flow; Crs, static respiratory system compliance; Ccw, chest wall compliance; Clung, lung compliance; EELV, end-expiratory lung volume measured by CT scan; MAP, mean arterial pressure; HR, heart rate; PAPm, mean pulmonary arterial pressure; PCWP, pulmonary capillary wedge pressure; CO, cardiac output; CVP, central venous pressure; SvO_2_, mixed venous saturation; BE, base excess; Lac, plasma lactates. Significant p-values are presented in bold characters*.

As respiratory rate decreased and ECMO CO_2_ extraction increased, mechanical power decreased (*p* < 0.001) in comparison to the RR 24 Baseline value (Table 2). At lower respiratory rates, plateau pressure (*p* < 0.001) and mean airway pressure (*p* = 0.021) slightly increased, while respiratory system compliance decreased (Table 2). Partitioned mechanics showed that lung compliance remained stable at increasing level of CO_2_ removal by ECMO, while chest wall compliance decreased (Table 2).

Finally, despite stable systemic arterial pressure and cardiac output, mean pulmonary artery pressure increased at lower RR (*p* < 0.05; Figure 3C and Table 2).

### Determinants of Physiological Impairments

In an effort to more precisely identify the mechanisms underlying poorer respiratory physiology, we explored correlations between physiologic changes induced by lower respiratory rate and higher ECMO CO_2_ extraction and the abovementioned observed effects.

**Figure 4:**
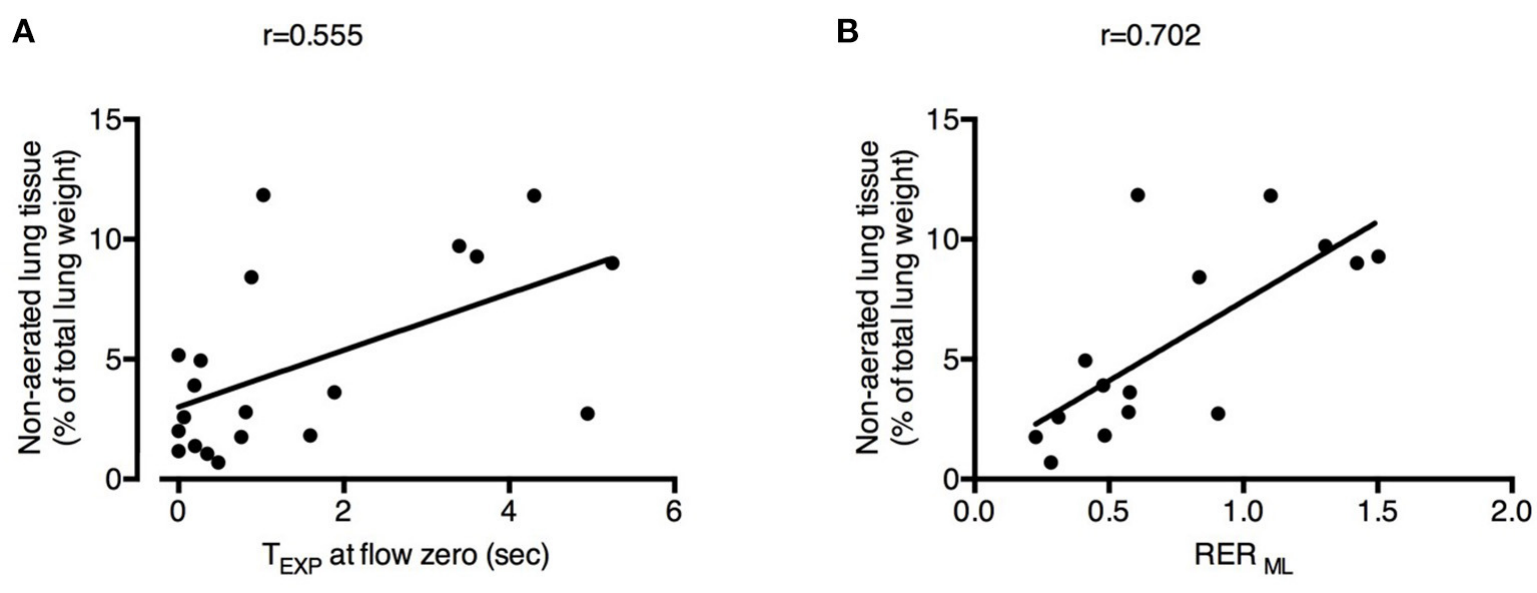
Physiological changes induced by decreased respiratory rate and increased extracorporeal CO_2_ removal associated with the development of atelectasis. Increased expiratory time spent at zero flow (T_EXP_ at flow zero) **(A)** and higher Respiratory Exchange Ratio of the membrane lung (RER ML) **(B)** were correlated with higher fraction of non-aerated lung tissue (i.e., atelectasis). See text for methodological details. Number of observations were *n* = 20 in **(A)** due to unavailability of calculation of expiratory time at flow zero in one of the animals and *n* = 15 in **(B)** due to impossibility of blood withdrawal to calculate RER ML in one of the animals.

The percentage of non-aerated lung tissue was correlated with the expiratory time spent at zero flow (*r* = 0.555; Figure 4A) and not with the mean or the plateau airway pressure (*r* = −0.333 and *r* = 0.102, respectively). The percentage of non-aerated lung was correlated also with the RER _ML_ (*r* = 0.702; Figure 4B), indicating that development of atelectasis may be more likely when ECMO is predominantly used to remove CO_2_.

**Figure 5:**
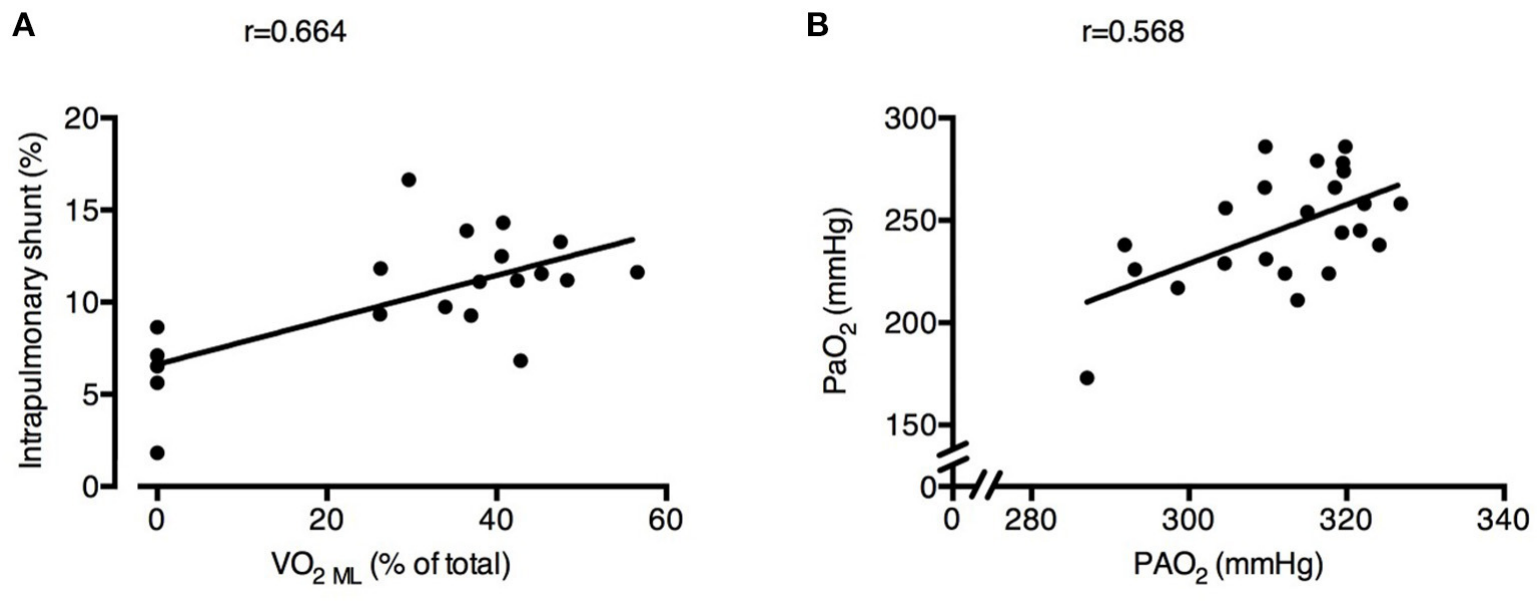
Physiological changes induced by decreased respiratory rate and increased extracorporeal CO_2_ removal associated with increased intrapulmonary shunt and poorer arterial oxygenation. Higher oxygen transfer by the ECMO membrane lung (VO_2_ ML) was correlated with larger intrapulmonary shunt fraction **(A)**. Alveolar O_2_ pressure (PAO_2_) was correlated with systemic arterial partial pressure of O_2_ **(**PaO_2_) **(B)**. See text for methodological details. Number of observations were *n* = 20 in **(A)** due to impossibility of blood withdrawal to calculate VO_2_ML in one of the animals and *n* = 23 in **(B)** due to blood gas analyzer failure for one arterial sample.

Intrapulmonary shunt was correlated with the SvO_2_ and with the % of VO_2_ granted by ECMO (*r* = 0.419 and *r* = 0.664, respectively; Figure 5A). Increasing ECMO CO_2_ extraction decreased the RER _NL_ (Baseline 0.74 ± 0.09 vs. RR18 0.87 ± 0.11 vs. RR12 0.78 ± 0.12 vs. RR6 0.46 ± 0.13, *p* < 0.001), resulting in lower alveolar PAO_2_ (RR24 312 ± 6 mmHg vs. RR18 321 ± 4 mmHg vs. RR12 315 ± 7 mmHg vs. RR6 300 ± 12 mmHg; *p* < 0.001). Systemic PaO_2_ was significantly correlated with PAO_2_ (*r* = 0.568; Figure 5B).

## Discussion

Study main findings are that progressive decrease of respiratory rate coupled with increased extracorporeal CO_2_ removal by ECMO leads to reduced mechanical power, but it is also associated with development of atelectasis, higher intrapulmonary shunt, and lower oxygenation in a large animal model of ECMO with healthy lungs. Development of atelectasis may be caused by longer motionless expiratory time (zero flow during expiration) and predominance of CO_2_ removal over oxygen delivery by the ECMO membrane lung. Increased shunt, evident already during the RR 18 phase, instead, may be correlated to oxygen transfer by ECMO, leading to higher mixed venous saturation. Finally, poorer systemic oxygenation may be caused by lower RER of the natural lung, yielding reduced alveolar O_2_ tension.

Seminal ECMO studies proposed reduction to very low frequency ventilation (3–5 breaths per minute) as optimal strategy for lung rest (Gattinoni et al., 1986). The two most recent randomized clinical trials on ECMO in severe ARDS applied much higher rates: fixed 10 breaths per minute in the CESAR trial (Peek et al., 2009) and an average of 23–24 breaths per minute in the more recent EOLIA study (Combes et al., 2018), leaving equipoise and clinical uncertainty. A study in pigs with acute lung injury described that rate of ~14 breaths per minute during extracorporeal CO_2_ removal was associated with lower levels of systemic and pulmonary inflammatory mediators in comparison to standard mechanical ventilation with ~30 breaths per minute (Grasso et al., 2014). However, the two ventilation strategies were applied only for 3 h. A more recent experimental study on animal model of ARDS compared different ventilation strategies for 24 h during ECMO. The one termed “near-apneic” with a rate of 5 breaths per minute showed decreased histologic lung injury score in comparison to other strategies with higher RR. However, the “near-apneic” strategy was also associated with impaired respiratory mechanics (Araos et al., 2019). Thus, physiological targets guiding the decrease of respiratory rate after ECMO start remain an open issue.

The aim of mechanical ventilation during ECMO is to decrease the risk of VILI (Marini et al., 2020), and reduction of respiratory rate is usually a key component of this strategy (Pesenti et al., 1982; Gattinoni et al., 1986; Peek et al., 2009; Terragni et al., 2009; Bein et al., 2013; Combes et al., 2018, 2019). As expected, in the present study, respiratory rate was associated with significantly decreased *mechanical power*, with larger reduction at lower rates, potentially suggesting linear correlation between reduced respiratory rate and lung protection (at constant inspiratory flow). However, progressive decrease of respiratory rate (especially at values lower than 12 breaths per minute) was also associated with development of atelectasis. Atelectasis may trigger two key mechanisms of VILI, potentially outweighing the beneficial effects of decreased mechanical power, namely, reduced baby lung size (i.e., the normally aerated lung fraction) causing increased *lung strain* (Bellani et al., 2011) and larger fraction of lung units opening and closing during the respiratory cycle (*atelectrauma*) causing additional local stress by the sudden diffusion of gas flow between the epithelial cells (Caironi et al., 2010). A larger fraction of atelectasis occurred in the dependent lung, suggesting that this region may be particularly prone to these detrimental mechanisms, as shown by a previous publication (Scaramuzzo et al., 2020b).

Reduced respiratory rate after ECMO start was also associated with increased intrapulmonary shunt and lower arterial oxygenation, confirming previous observations in ARDS patients (Fanelli et al., 2016; Spinelli et al., 2020). Higher shunt and poorer oxygenation may not be detrimental *per se* for the lungs. However, in clinical practice, they could lead to use of higher PEEP levels and more aggressive recruitment during ECMO and, in turn, with increased risk of *overdistension* and *barotrauma* (Mauri et al., 2016a), further increasing the risk of undermining the benefits of reduced mechanical power.

We described increased pulmonary artery pressure at lower respiratory rate that may stress the right heart function, which is a major determinant of ARDS outcome (Mekontso Dessap et al., 2016).

In summary, our data indicate that progressive decrease of respiratory rate after ECMO start is associated with lung-protective effects but may place the basis for mechanisms potentially promoting VILI.

Deeper understanding of the mechanisms that caused the abovementioned physiological effects may be key to tailor personalized ventilation and ECMO settings. A previous study showed that expiratory time longer than 4 s promotes lung collapse in a large animal model of ARDS (Neumann et al., 1998). Our results are in line with those findings: expiratory time was 6.7 s during the RR 6 phase (the one associated with larger fraction of atelectasis) vs. 3.3 s during RR 12 and 2.2 s for RR 18. We also described a more direct association between the development of atelectasis and the “no motion” time at end expiration. This phenomenon is probably due to lack of fresh gas replacement favoring reabsorption and it seems reasonable from a physiological point of view as collapse more likely occurs when the alveolar pressure reaches its lowest level (Duggan et al., 2005). Larger fraction of atelectasis was also correlated with predominance of CO_2_ extraction over O_2_ delivery by the ECMO membrane lung: this might be due to collapse of instable lung units with a low ventilation–perfusion ratio, which may have increased at lower respiratory rates by reduced alveolar ventilation. Indeed, these units are more prone to collapse due to the shift of alveolar nitrogen to relatively denitrogenated venous blood (Dantzker et al., 1975). Interestingly, shorter expiratory time spent at zero flow would not influence the latter mechanism and atelectasis would develop anyway, while a lower concentration of oxygen in the ECMO sweep gas flow might be protective.

We showed that increased intrapulmonary shunt was proportional to oxygen delivery by ECMO and to mixed venous saturation: blunting of hypoxic vasoconstriction by higher oxygenation of venous blood is known to increase shunt and could be the underlying mechanism (Spinelli et al., 2020). In the future, personalized ECMO settings might be aimed at minimizing these effects.

Finally, arterial and alveolar O_2_ tensions were correlated in our study, as expected in healthy lungs (Riley and Cournand, 1949). Decreased arterial and alveolar PO_2_ at lower respiratory rates were caused by lower RER of the natural lung due to higher CO_2_ extraction by ECMO. Of note and as previously described, the RER of the natural lung decreased only at very high CO_2_ extraction rate by ECMO and relatively low FiO_2_ at the ventilator and may not be particularly relevant during standard clinical use (Abrams et al., 2020).

Personalized respiratory rate in ARDS patients on ECMO may be chosen as the one associated with reduced mechanical power without increase in atelectasis. For example, continuous dynamic EIT monitoring could identify the lowest respiratory rate avoiding decrease of the dependent lung ventilation. On average, among the respiratory rates explored in this study, 12 breaths per minute was associated with decreased mechanical power and minimal detrimental effects.

This study has relevant limitations in the design of the experiment. First, the respiratory rates were not randomized since, to increase the clinical impact, we decided to assess the detrimental effects of progressive decrease of respiratory rate and not to compare selected target respiratory rates, which would have been anyway debatable. The development of atelectasis likely occurred by interaction of decreased rate and time under controlled mechanical ventilation and paralysis. The progressive decrease in chest wall compliance might be a confounding factor, possibly due to surgical procedures, and time under paralysis and anesthesia, while the lack of change in lung compliance with decreasing RR might be explained by the relatively small amount of atelectasis. Second, tidal volume was left unchanged during the study, while it is usually decreased after ECMO start in the clinical practice reduction. However, further hypoventilation due to reduced tidal volume might have amplified the unphysiological effects that we measured (e.g., atelectasis). Third, some ECMO centers increase PEEP in severe ARDS patients after ECMO start (Schmidt et al., 2019) to limit de-recruitment while we left unchanged low PEEP level of 5 cmH_2_O. Higher PEEP might counteract the decrease in EELV due to atelectasis. Fourth, the study was performed exclusively on healthy animals, which limits clinical significance, but our choice is intended to remove the problem of discriminating between hypoventilation-induced atelectasis and loss of aeration due to lung injury and edema, which are predominant mechanisms leading to loss of aeration in ARDS.

## Conclusions

In the end, our study is an exploratory investigation to test the physiological effects of reduced respiratory rate *per se* during ECMO. Progressive decrease of respiratory rate and increased CO_2_ extraction in a large animal model of ECMO with healthy lungs are associated with not only decreased mechanical power but also development of atelectasis, higher intrapulmonary shunt, and lower arterial oxygenation. We observed mild but potentially detrimental physiological effects that deserve attention in order to personalize optimal ventilation and ECMO interaction guaranteeing protective respiratory mechanics and minimal adverse events.

## Data Availability Statement

The raw data supporting the conclusions of this article will be made available by the authors, without undue reservation.

## Ethics Statement

The study was approved by the Italian Ministry of Health (protocol n. 749/2019) and conducted according to the European Directive 2010/63/EU on the protection of animals used for scientific purposes and Italian legislative decree 26/2014. The research protocol was approved by the Institutional Animal Care Committee.

## Author Contributions

ESp, ESc, LR, SG, and TM: substantial contributions to the conception or design of the work. ESp, GC, GD, ESc, IM, EG, AM, DD, RM, MB, OB, and TM: acquisition, analysis, interpretation of data for the work, and agreement to be accountable for all aspects of the work in ensuring that questions related to the accuracy or integrity of any part of the work are appropriately investigated and resolved. All authors: drafting the work or revising it critically for important intellectual content and final approval of the version submitted for publication.

## Conflict of Interest

TM received personal fees from Fisher & Paykel, Drager, Mindray, and BBraun outside of the present work. The remaining authors declare that the research was conducted in the absence of any commercial or financial relationships that could be construed as a potential conflict of interest.

## Abbreviations

VILI: ventilator-induced lung injury
IV: intravenous
RR: respiratory rate
Crs: respiratory system compliance
Clung: lung compliance
Ccw: chest wall compliance
RER: respiratory exchange ratio
MAP: mean arterial pressures
PAPm: mean pulmonary artery pressures
WP: wedge pressure
BE: base excess
NL: natural lung
EIT: electrical impedance tomography
BF: blood flow
GF: gas flow
ML: membrane lung.
